# Fecal Shedding, Antimicrobial Resistance and *In Vitro* Biofilm formation on Simulated Gallstones by *Salmonella* Typhi Isolated from Typhoid Cases and Asymptomatic Carriers in Nairobi, Kenya

**DOI:** 10.14302/issn.2690-4721.ijcm-24-5030

**Published:** 2024-04-24

**Authors:** Peter Muturi, Peter Wachira, Maina Wagacha, Cecilia Mbae, Susan Kavai, Musa Muhammed, John S. Gunn, Samuel Kariuki

**Affiliations:** 1 Centre for Microbiology Research, Kenya Medical Research Institute; 2 Department of Biology, University of Nairobi, Kenya; 3 Ministry of Health, Kenya; 4 Center for Microbial Pathogenesis, Abigail Wexner Research Institute at Nationwide Children's Hospital, Columbus, OH, USA; 5 Infectious Diseases Institute, The Ohio State University, Columbus, OH, USA; 6 Wellcome Sanger Institute, Cambridge, United Kingdom; 7 Drugs for Neglected Diseases initiative Eastern Africa, Nairobi, Kenya

**Keywords:** *Salmonella*, biofilm, AMR, antibiotics, typhoid

## Abstract

Typhoid fever, caused by the human restricted pathogen *Salmonella* Typhi, remains a major global public health concern. Even after successful treatment, approximately 3–5% of patients with typhoid fail to clear the bacteria within one year and become chronic carriers. Most typhoid carriers have gallstones in their gallbladder, and biofilm formation on gallstones is highly correlated with chronic carriage. This study’s goal was to identify asymptomatic typhoid carriers in an endemic setting in Kenya, and to compare acute versus chronic isolates. A cohort of typhoid fever patients identified through blood and/or stool culture, and their household contacts, were followed up after treatment to detect longitudinal *S*. Typhi stool shedding. An abdominal ultrasound scan was used to identify individuals with gallstones. A total of 32 index patients and 32 household contacts were successfully followed-up. Gallstones were detected in 4 cases and 1 household contact. The duration of *S*. Typhi shedding was significantly longer in individuals with gallstones compared to those without, *P*<0.001. Eighty-three (83) *S*. Typhi strains were tested for susceptibility to commonly used antimicrobials and examined by *in vitro* biofilm formation assays. Out of 37 infected individuals, 32.4% had infections caused by multidrug resistant (MDR) *S*. Typhi strains and only 18.9% were infected by susceptible strains. Non-MDR strains formed significantly better biofilms *in vitro* than the MDR strains (*P*<0.001). This study provides data on *S*. Typhi chronic carriage that will influence public health approaches aimed at reducing typhoid transmission and the burden of infection.

## Introduction

*Salmonella enterica* serovar Typhi (*S.* Typhi) is a human-restricted pathogen, and causes both acute systemic infections (typhoid fever) and chronic infections (with asymptomatic carriage) (1,2). Although it has largely been eliminated in high income countries, typhoid (enteric) fever remains a major global public health concern especially among low- and middle-income countries (LMICs) (3), with an estimated annual global incidence of 9 million cases, and ~110,000 fatalities per year (4). Risk of transmission is highest in densely populated areas with poor sanitation and inadequate safe drinking water (5,6). *S.* Typhi can persist in external environment, but with a limited capacity to multiply outside the human host, and can spread within communities through contamination of food and water in the immediate vicinity of the case (short-cycle) or carriers (7,8). Although highly contagious, carriers are typically asymptomatic and shed the infectious bacteria intermittently in their feces for an ill-defined period of time and thus may spread the disease and serve as a reservoir of infection (1).

With appropriate antibiotic treatment, most patients resolve the disease but approximately 3–5% fail to clear the bacteria within one year, and therefore are defined as chronic carriers. The asymptomatic carriage of *S*. Typhi has been recognized as a public health threat for over a century with the infamous carriers like Mary Mallon (Typhoid Mary), a cook in New York identified in the early part of 20^th^ century (9). In chronic carrier state, *S.* Typhi colonizes the biliary tract and approximately 90% of chronically infected carriers have GSs in their gallbladder (1,10,11). Presence of *S.* Typhi biofilms on cholesterol-rich gallstones of typhoid carriers has been demonstrated (12,13). The biofilms are usually enveloped in bacteria-initiated matrix (extracellular polymeric substances) allowing the organisms to survive in hostile conditions including exposure to salinity, phagocytes, and several antimicrobials (14). This makes antibiotics ineffective in clearing *S.* Typhi bacteria from the gallstones making gallbladder removal (cholecystectomy) the only effective therapy (11).

Inexpensive and readily available antibiotics including streptomycin, ampicillin, chloramphenicol and trimethoprim-sulfamethoxazole are becoming ineffective or suboptimal for treating typhoid due to the emergence of multidrug-resistant (MDR) strains (15), mainly by the dominant Haplotype 58 (genotype 4.3.1) clade of *S.* Typhi (3). In addition, increased fluoroquinolone resistance has become common in South-East Asia and has sporadically been reported in sub-Saharan Africa (16–18).

Assessing and understanding the pattern of shedding of *S*. Typhi, especially in asymptomatic carriers living in endemic typhoid settings is essential to influence public health approaches aimed at reducing disease transmission and burden of infection (19). The current study aimed at understanding the role of asymptomatic typhoid carriers in transmission of *S*. Typhi at the household level in an urban endemic setting in Nairobi, Kenya and the relationship between persistent infection, duration of shedding, presence of gallstones, antimicrobial resistance, and *in vitro* biofilm formation.

## Materials and Methods

### Study Site and Participants

This study involved following up blood and/or stool culture-positive typhoid cases from four primary health centers in Mukuru kwa Njenga and Mukuru kwa Ruben informal settlements, an endemic typhoid setting in Nairobi, Kenya (20), and Mama Lucy Kibaki hospital, a Level-5 county referral hospital in Nairobi, between December 2020 and November 2023. The level-5 hospital is situated approximately 10 Km from Mukuru and serves the population in most neighborhoods in the Eastern part of Nairobi including the Mukuru informal settlements.

Patients who were ≥12 years old presenting with symptoms of typhoid fever were consented to take part in the carriage study, and each was requested to provide their phone number and that of the head of the household. Blood and stool samples were collected and transported to microbiology laboratory at the Centre for Microbiology Research, Kenya Medical Research Institute (KEMRI) for culture and antimicrobial susceptibility testing (AST). *S.* Typhi culture-positive samples were identified using biochemical tests on Analytical Profile Index 20E (API20E) and by Polymerase Chain Reaction (PCR). Respective patients’ household addresses were located through the help of a community health worker (CHW) living in the same neighborhood as the index case. The household head was contacted to explain about the study and household members ≥12 years old were invited to participate. Global Positioning System (GPS) coordinates of the household address were taken using Epicollect data collection tool and used to map hotspots for *S.* Typhi carriage.

### Detection of *S.* Typhi Shedding

*Salmonella* positive patients were offered treatment, with all taking various antibiotics and durations of the medications (Supplementary Table S1). After completion of the course of antibiotics, stool samples were collected with the aim of starting follow-up cultures 14 days after diagnosis/start of treatment. Proposed dates of sample collection were, however, affected by availability of the index case and their household members. Each study participant was provided with a stool container with a unique identifier on the day of sampling and it was explained to them how to collect the stool sample. To ensure optimal recovery of the pathogen, a sterile swab was used to aliquot part of the specimen in Cary Blair Transport Medium (Oxoid Ltd., Basingstoke, UK) before packing in a zip lock bag, which was then placed in a cooler box (2–8°C) and transported to the laboratory at KEMRI. The samples were collected at least three times during the first month, and once monthly thereafter. The goal was to monitor shedding of *S*. Typhi for 12 months.

### Stool Culture, Identification and Confirmation of *Salmonella* Typhi Isolates

All samples from index cases and household contacts were initially cultured in selenite F broth (Oxoid, Basingstoke, UK) within 4 h of collection. Laboratory processing was as previously outlined (20). Briefly, broth containing stool samples was incubated aerobically overnight at 37°C before subculturing on Xylose Lysine Deoxycholate (XLD) and MacConkey (Oxoid) agar plates. The plates were incubated aerobically at 37°C overnight. Non-lactose fermenting colonies were characterized phenotypically using standard biochemical testing (Triple sugar iron (TSI), Urea and Motility), and the Analytical Profile Index (API System, Montalieu Vercieu, France). The Kauffmann–White classification scheme was used in serogrouping and identification of isolates suspected to be *S*. Typhi using monovalent antisera (Murex Diagnostics, Dartford, UK). All isolates were archived in a −80°C freezer until further analysis.

### Antimicrobial Susceptibility Testing

Antimicrobial susceptibility testing was performed using the disk diffusion technique for all commonly used antimicrobials in Kenya including ampicillin (10 μg), tetracycline (30 μg), co-trimoxazole (25 μg), chloramphenicol (30 μg), amoxicillin-clavulanate (20/10 μg), cefpodoxime 30 μg, ceftazidime (30 μg), ceftriaxone (30 μg), cefotaxime (30 μg), azithromycin (15 μg), ciprofloxacin (5 μg), nalidixic acid (10 μg), kanamycin (30 μg) and gentamicin (10 μg). The diameter of the zone of inhibition was measured and results were interpreted according the Clinical and Laboratory Standards Institute (CLSI) guidelines for *Salmonella* (CLSI 2022, 2023).

### Gallstone Identification on Suspected *Salmonella* Typhi Carriers

Ultrasonography (US), the primary imaging modality performed by a radiologist used to evaluate patients suspected of having gallbladder disease, was used to demonstrate gallstones or gallbladders with a thickened wall. The scans were performed in the morning of the agreed day before the study participant ate breakfast, and thus before bile was released from the gallbladder.

### Detection of *Salmonella* Typhi by PCR

A pure colony of bacteria grown overnight on Luria-Bertani agar (LBA) was placed into a PCR tube containing 30 μL PCR mix consisting of 3.0 μL 10 × PCR reaction buffer, 0.3 μL Taq DNA polymerase, 1.2 μL 50mM MgCl_2_, 0.6 μL dNTPs and 24 μL ultra-pure DNAse/RNAse free water (Invitrogen; Thermo Fisher Scientific; Hampton, NH). Primers (each 0.3 μL) were as follows: *tvi*B-F, 5′-TCAGCGACTTCTGTTCTATTCAAGTAAGAAAGGGGTACGG-3′ and *tvi*B-R, 5′-GCTCCTCACTGACGGACGTGCGAACGTCGTCTAGATTATG-3′. The primers were used to amplify a 1278 bp fragment of the VI region of the flagellin gene. Amplification was carried out using a PCR machine (The Mastercycler^®^ nexus X2; Eppendorf; Hamburg, Germany) in stages, with an initial denaturation at 94°C for 4 minutes. A further 25 cycles were then carried out, each cycle comprising denaturation at 94°C for 15 seconds, annealing at 58°C for 30 seconds, then extension at 72°C for 1 minute 30 seconds, followed by (after the final cycle) 72°C for 10 minutes.

### *in vitro* Biofilm Formation Assays

*S.* Typhi biofilms were grown on non-treated polystyrene 96-well plates (Corning, Kennebunkport, ME). To simulate growth conditions on gallstones, wells in two plates were pre-coated with cholesterol by adding a solution of 5mg/mL in 1:1 isopropanol:ethanol and air-dried overnight. A pure colony of *S*. Typhi on an XLD agar plate was cultured in Tryptone Soy Broth (TSB). Overnight (O/N) cultures in broth were normalized to OD_600_=0.8, diluted 1:2500 in TSB or TSB containing 2.5% human bile, and 100 μL/well were dispensed into the plates. The plates were incubated at 25°C in a Fisherbrand^™^ nutating mixer (Thermo Fisher Scientific; Hampton, NH) at 24rpm for 96 hours. Media (TSB or TSB containing bile) was changed after every 24 hours for consistent *S.* Typhi biofilm growth. Plates were emptied on the 4^th^ day and washed twice before heat fixing at 60°C for 1 hour. The biofilms were stained using a crystal violet solution. Acetic acid (33%) was used to elute crystal violet before reading the OD_570_.

#### Statistical Analysis

Statistical analysis was performed using GraphPad Prism 9.0. Analysis of variance (ANOVA) was used to test level of significance in biofilm formation between any three independent groups, i.e., biofilms in absence of cholesterol and bile, in cholesterol coated plates in absence of bile, and in presence of cholesterol and bile. For biofilm formation by antibiotic non-resistant strains, quinolone non-susceptible and multidrug resistant *S.* Typhi, P-values less than 0.05 (P<0.05) were considered significant. An unpaired Student’s t-test was used to compare the means of two independent groups including duration of shedding by individuals with gallstones vs. those without, biofilm formation by isolates from these two groups, as well as multidrug resistant vs. non-MDR *S*. Typhi strains.

## Results

### Study Participants and Sample Collection

**Figure 1.**
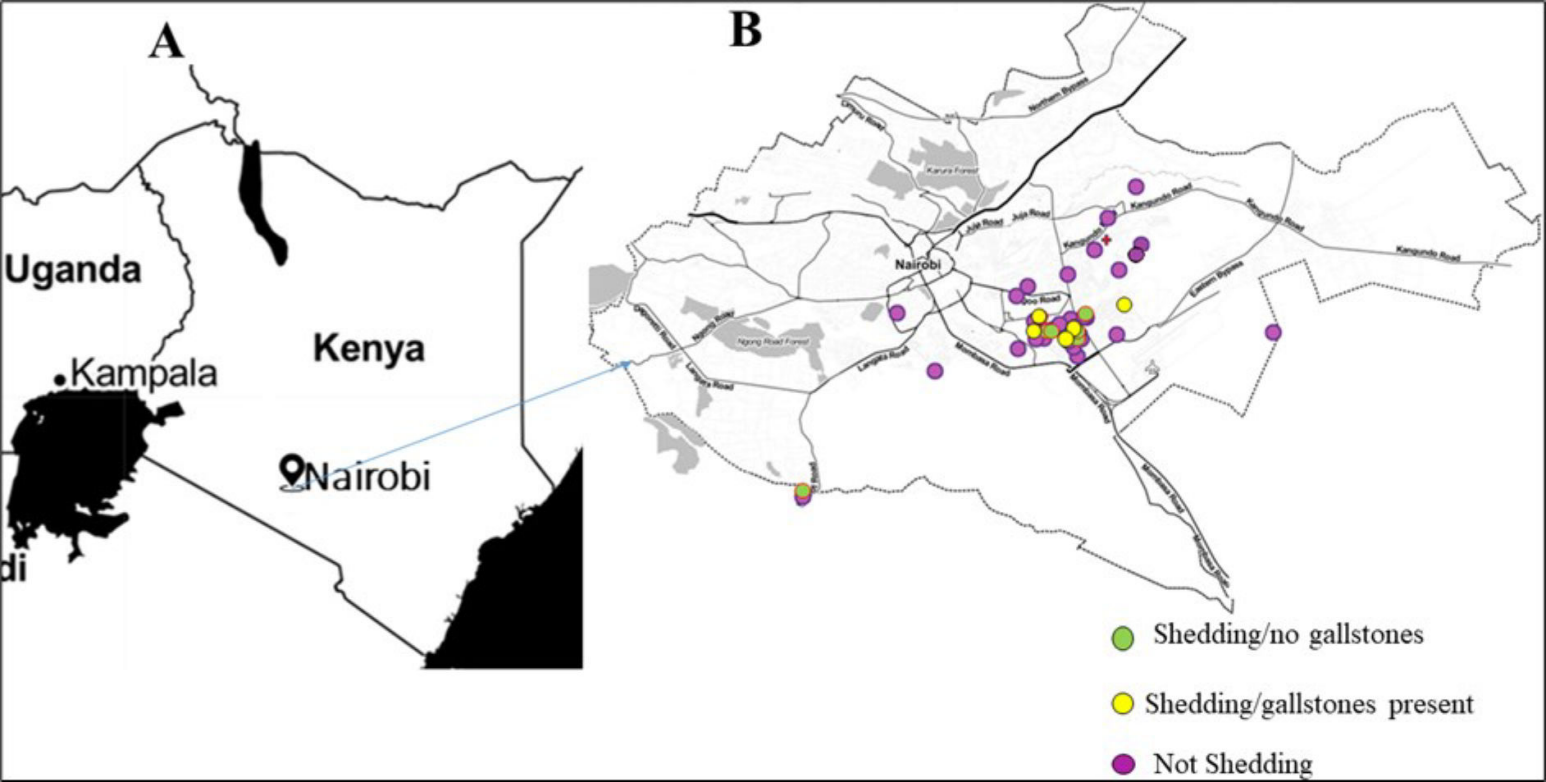
Study area. (A) Map of Kenya showing the location of Nairobi. (B) Geographical distribution of typhoid index cases and asymptomatic carriers in Nairobi.

Between December 2020 and November 2023, a total of 2,792 patients ≥12 years old with typical symptoms of typhoid fever were recruited, and both blood and stool collected for culture. The total number of culture positive cases was 42 (1.5% positivity rate). A total of 32 typhoid index cases were successfully followed-up after treatment, representing 76.1% of all patients diagnosed with typhoid fever. The median age of followed up culture positive cases was 29.4 (12.2–56.9 years). Male and female participants were 50% each. Geographical distribution of index cases is as shown in Figure 1. After obtaining consent and depending on the number of people ≥12 years old living with the index case in the household, a maximum of three contacts were invited to participate. The median age of the household contacts (n=32) was 28.2 (12.9–49.7 years), 57.5% male and 42.4% female, (Supplementary Table S2). Stool samples were collected for varying durations depending on the willingness of participants to continue taking part in the study and availability, as some study participants relocated to other parts of the country before completion of follow ups.

### *Salmonella* Typhi Shedding

Shedding was observed in 9/32 (28.1%) of the index cases and 5/32 (15.6%) of household contacts. All of these, 14 study participants, shed *S.* Typhi during the first and/or the second month, with only 4 shedding after the second month (Table 1). In 3/32 (9.3%) of the households, the index case and one household contact shed the bacteria. The pathogen was shed only by the index case in 6/32 (18.7%) of the households and by a household contact only in 2/32 (6.2%) of the households. A total of 5 study participants, 4/32 (12.5%) of index cases and 1/32 (3.1%) of household contacts, were found to have gallstones in their gallbladder. Thus, of the 14 patients shedding the bacteria in follow-up visits, 5 had gallstones. Although no shedding was detected past the sixth month, the duration of shedding was longer in individuals with gallstones [(M=115.6, SD=48.67) vs. without gallstones (M=31.78, SD=11.18) *P*=0.003)].

### Antibiotic Resistance Testing

A total of 83 *S.* Typhi strains were tested for susceptibility to antimicrobials. As shown in Table 2, majority, 11/32 (34.3%) of the index cases were infected by *S.* Typhi strains with reduced susceptibility to ciprofloxacin and showing resistance to ampicillin, co-trimoxazole, chloramphenicol and nalidixic acid. A substantial 7/32 (21.8%) of the cases were infected with *S*. Typhi that was pan-susceptible, while 3/32 (9.3%) were infected by non-MDR *S*. Typhi strains although showing resistance to nalidixic acid and reduced susceptibility to ciprofloxacin. Other observed resistance patterns include non-MDR strains with reduced susceptibility to both nalidixic acid and ciprofloxacin (infecting 5/32 [15.6%] of typhoid index cases) and non-MDR strains with reduced susceptibility to ciprofloxacin (infecting 2/32 [21.8%] of typhoid index cases). Among the cases shedding the bacteria after treatment, 4/19 (44.4%) shed MDR strains of *S*. Typhi. Isolates from persistently infected household members also had varying resistance patterns, as 1/5 (20%) shed strains with reduced susceptibility to ciprofloxacin and showing resistance to ampicillin, co-trimoxazole, chloramphenicol and nalidixic acid. One persistently infected household member shed *S*. Typhi strains resistant to nalidixic acid and with reduced susceptibility to ciprofloxacin, while 2/5 (40%) shed strains with reduced susceptibility to both nalidixic acid and ciprofloxacin, and 1/5 (20%) shed *S*. Typhi with reduced susceptibility to ciprofloxacin (Table 2). *S.* Typhi strains isolated before and after treatment showed similar patterns of antimicrobial resistance in 13/14 (92.8%) of the study participants. However, one index case initially infected by an *S*. Typhi strain that was susceptible to all tested antimicrobials, shed an *S.* Typhi showing reduced susceptibility to ciprofloxacin 54 days later. Finally, 4/5 (80%) of the patients with gallstones shed non-multidrug resistant *S*. Typhi.

### Biofilm Formation

**Figure 2.**
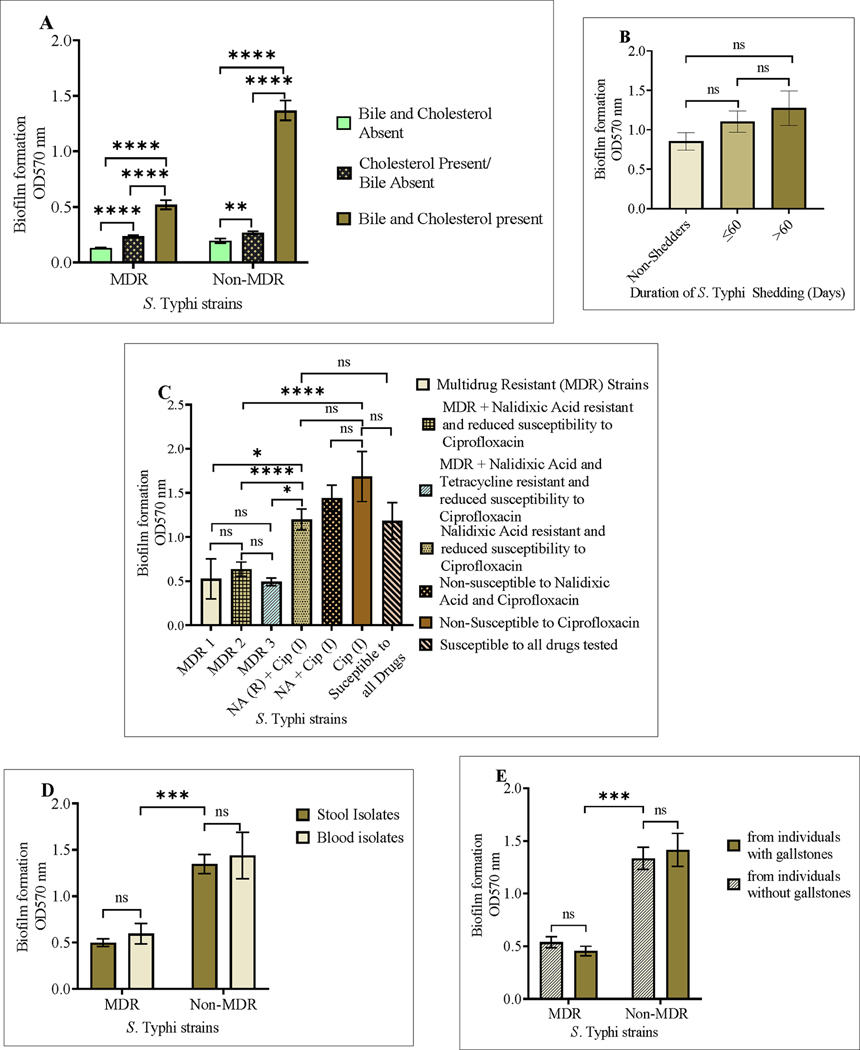
*S.* Typhi biofilms. (A) Quantity of biofilms after growth in presence/absence of cholesterol and/or bile. (B) Biofilm formation by *S*. Typhi isolated from non-shedders, patients shedding for one and/or two months (≤ 60days) and from patients shedding the bacteria during and/or after the third month (>60 days). (C) Biofilm formation by MDR and non-MDR strains. (D) MDR and non-MDR strains isolated from blood vs. stool, and (E) strains isolated from individuals with gallstones vs. participants without gallstones. Error bars represent SEM, ****, *P*<0.001; ***, *P*<0.005; **, *P*<0.05; ns, *P*>0.5.

Because of the importance of biofilm formation on gallstones in chronic carriage (11,21) the biofilm forming ability of human *S*. Typhi isolates were tested under gallbladder simulating conditions. Variations were observed in the quantity of biofilm mass produced by the 83 *S.* Typhi strains. All isolates formed weak biofilms in wells without cholesterol and human bile, however, they formed significantly stronger biofilms in presence of both cholesterol and bile (*P*<0.001, Figure 2A).

Comparisons on biofilm forming ability between the different isolates was therefore based on results obtained after growing the biofilms in presence of human bile in cholesterol coated plates. From those individuals shedding *S*. Typhi, there was no statistical association between duration of *S*. Typhi shedding and biofilm forming ability (Figure 2B). As shown in Figure 2C, significant differences were noted in biofilm mass quantities, whereby the MDR strains formed relatively weak biofilms (approx. OD_570_=0.5) as compared to both susceptible and quinolone non-susceptible strains (approx. OD_570_=1.3), (F [2, 29] = 30.96, *P*=<0.001). Additionally, there was no statistically significant association between biofilm forming ability and the sample location (stool or blood), for both MDR and non-MDR strains (*P*>0.05, Figure 2D). Similarly, in those shedding the bacteria, the presence or absence of gallstones did not affect the biofilm mass formed (*P*>0.05, Figure 2E).

## Discussion

In this study, longitudinal *S.* Typhi shedding was observed in 9/32 (28.1%) of typhoid index cases even after treatment with appropriate antibiotics. Shedding was also observed in 5/32 (15.6%) asymptomatic individuals living in the same household with typhoid index cases, indicating the presence of *S.* Typhi reservoirs, intermittently shedding and transmitting the pathogenic bacteria within the household. A similar study conducted in South India identified asymptomatic *S.* Typhi carriers living in same household with children diagnosed with typhoid fever (8). Since there is no known or suspected animal reservoir for *S*. Typhi, such asymptomatic carriers allow persistence of the disease during inter-epidemic periods (22), leading to the spread of infections through the fecal-oral route of transmission. These contribute to the global burden of typhoid fever, where the adjusted global incidence is 134.1/100,000 person-years (95% CI, 77.9–204.8) (23). In Sub-Saharan Africa, Central Africa has the highest reported incidence of typhoid fever at 1459 per 100,000 person-years, while East Africa has the second highest incidence of 620 per 100,000 person-years (24). Cases tend to be concentrated in younger age groups in settings with high transmission (25,26), where major burden of typhoid fever is borne by preschool and school-aged children (22). Water, sanitation, and hygiene (WaSH)-related enteric diseases are endemic in our study setting (20), with a previous study reporting an observed case culture positive rate of 4.3% among febrile children 7–16 years of age and a substantial burden among younger children 1–7 years of age (3.1 positive rate) and infants (culture positive rate of 2.2%).

The majority of index cases, 11/32 (34.3%), were infected by strains showing resistance to four antimicrobials (ampicillin, co-trimoxazole, chloramphenicol and nalidixic acid) and associated with reduced susceptibility to ciprofloxacin. Some of these cases, 4/32 (12.5%) continued to shed MDR strains of *S*. Typhi even after treatment, therefore spreading resistant bacteria within the household, and possibly extending transmission to other members of the community. Out of the five study participants with gallstones (they shed *S*. Typhi for longer duration), 1/5 (20%) shed susceptible strains, 1/5 (20%) shed MDR strains while 3/5 (60%) continued to shed non-MDR strains associated with reduced susceptibility to ciprofloxacin. Cases of multi-drug resistance to conventional first line antibiotics, i.e., ampicillin, co-trimoxazole and chloramphenicol) strains of *S.* Typhi have been reported since the early 1970s (27). With the introduction of fluoroquinolones for the treatment of typhoid, nalidixic acid-resistant strains associated with reduced susceptibility to fluoroquinolones have been increasingly reported in recent years (28,29). Azithromycin which was first tested in the 1990s (30) and cephalosporins are now some of the alternative drugs for the treatment and control of such typhoid cases. The resistance patterns in strains from carriers reflects the pattern of resistance in *S*. Typhi circulating in our study setting, suggesting no single AMR status is associated with carriage. Shedding of MDR *S.* Typhi by asymptomatic carriers working in the food industry has previously been reported (31). While shedding in our study did not extend beyond 6 months for any patient, it is unclear if the organism was cleared or if there is just intermittent release of the bacteria, which has been previously noted in carriers (10). There is limited published data on resistance patterns in *S.* Typhi isolated from asymptomatic carriers, as most studies have so far focused on acute typhoid fever cases. Little progress in understanding the progression of *S*. Typhi infections has been made in the past decade, especially the mechanisms that catalyze carriage (11). Detection of chronic carriers utilizing serial stool culture and isolation of the bacteria is considered labor-intensive with limited data being generated in recent past.

Study participants with gallstones in our study were found to shed *S*. Typhi for significantly longer duration. Out of the 5 individuals with gallstones, 3 (75%) shed the bacteria for more than three months. Gallstones have been reported to facilitate chronic carriage of *S*. Typhi by biofilm formation on gallstone surfaces (11). During the process of colonization of the gallbladder, *S*. Typhi is exposed to bile, a complex digestive secretion containing bile acids, cholesterol, phospholipids and bilirubin. Bile possesses strong antimicrobial properties (32), and the ability of *S*. Typhi to tolerate this apparently hostile environment indicates that bile resistance plays a central role in acute and chronic pathogenicity and gallbladder infection (13). Strong biofilms were formed when both cholesterol and bile were present. Bile is considered an important factor that influences biofilm development (12,33). Risk factors for the development of gallstones are female sex, age, pregnancy, physical inactivity, obesity and overnutrition (34). Close to 10% of the human population have gallstones and therefore gallstone (which increases dramatically as one ages) disease is quite widespread (35). Some studies have reported isolation of contrasting genotypes of *S*. Typhi strains from the gallbladder of cholelithiasis patients undergoing cholecystectomy and blood samples of acute cases living within the same region (36). Besides facilitating *S*. Typhi carriage and transmission, *Salmonella* carriers with gallstones have an 8.47-fold-higher risk for developing cancer of the gallbladder (37), as persistent gallbladder infection increases the risk of epithelium damage by carcinogenic toxins produced by *S*. Typhi (38).

There was a non-significant increase in biofilm formation by individuals shedding *S*. Typhi over several months vs. those not shedding the bacteria, which does not corroborate previous findings by our group (39). However, our current study has only a limited number of “shedding” isolates that may not be from truly chronically infected individuals. However, *S.* Typhi did show varying ability to form biofilms on simulated gallstones, with the non-MDR resistant strains forming stronger biofilms compared to MDR strains. The mechanism behind this finding is not known and requires additional experimentation. The association between antibiotic resistance and biofilm formation noted in this study is different from what was observed in non-typhoidal *Salmonella* strains, where susceptible strains reportedly failed to form multicellular behavior (40), suggesting differences in biofilm regulatory mechanisms in non-typhoidal *Salmonella* vs. *S*. Typhi strains carrying different antimicrobial resistance determinants. Biofilms have been reported to protect *Salmonella* from killing by ciprofloxacin (15,41), a second-generation fluoroquinolone that is recommended first line agent for treating typhoid fever (42). In vivo studies on carriage mechanisms of the human-restricted pathogen *S*. Typhi have been hampered by the lack of a tractable small animal model, with in vivo data relying extensively on the murine model of infection with the related serovar, *S*. Typhimurium. This study provides a basis of studying genetic differences between *S*. Typhi strains with different antimicrobial resistant patterns and biofilm forming abilities.

This study is not without limitations. Firstly, most of the samples for detection of *S.* Typhi shedding were collected from a small region in Nairobi, and this may therefore not represent the overall antimicrobial resistance and shedding patterns in Kenya. Further, none of the patients shedding *S*. Typhi were followed-up to the point of cholecystectomy, and therefore we lack gallstones/bile culture isolates for comparison with those from stool samples. Finally, as mentioned previously, the limited number of isolates in some comparisons may affect the conclusions that are drawn, and the inability to detect carriage over one year hampers our capacity to define strains as chronic isolates.

## Conclusion

Data analyzed in this study shows a strong correlation between presence of gallstones and duration of *S.* Typhi shedding. Non-MDR strains analyzed were found to form stronger biofilms as compared to MDR strains. No single AMR status was observed to be associated with asymptomatic typhoid carriage. It is necessary to understand the role of *S.* Typhi asymptomatic carriers in the persistence, evolution and transmission of typhoid fever in endemic areas. This study provides important information that will influence public health approaches aimed at reducing transmission of typhoid fever and burden of infection.

## Supplementary Material

Supplementary Table S1

Supplementary Table S2

## Figures and Tables

**Table 1.** Patterns of *S*. Typhi shedding by typhoid index cases and household contacts

	Study Participant Shedding *S*. Typhi	Days between index case and follow-up sample collection											
		Month 1	Month 2	Month 3	Month 4	Month 5	Month 6	Month 7	Month 8	Month 9	Month 10	Month 11	Month 12
**H3**	Index Case	28	**31**, 38 46	61	94	-	153	-	-	-	-	-	-
H6	*Index Case	-	**31, 35, 39**, 60	**88**	-	143	172	-	214	241, 269	300	-	335
	Household Contact	-	**31**, 35, 39, 60	88	-	143	-	-	-	-	-	-	-
H7	Index Case	**14, 16, 19** 26	39	71	106	-	-	-	-	-	-	-	-
	Household Contact	16, **18**, 22, 29	43	91	-	137	-	-	-	-	-	-	-
H8	*Index Case	**22**, 25, 30	37, 51	**78**	-	-	**170**	203	239	268	-	303	-
**H9**	Index Case	**15**, 17, 21	**35**, 46	74	-	Day 133	-	-	-	-	-	-	-
H14	Household Contact	25	**31, 35, 42**	66	-	-	-	-	-	-	-	-	-
**H17**	*Index Case	**21, 24, 28**	**35**	**76**	**107**	-	-	-	-	-	-	-	-
H21	*Household Contact	26, **29**	**33, 40, 54**	82	-	138	-	-	-	-	-	-	-
H23	Index Case	-	40, 49, **54**	61, 75	-	-	-	-	-	-	-	-	-
H28	*Index Case	8, 19, 26	**40**	68	**96**	**124**	159	-	-	-	-	-	-
**H29**	Index Case	**23, 26, 30**	37, 51	-	-	-	-	-	-	-	-	-	-
	Household Contact	**23, 26**, 30	37,51	-	-	-	-	-	-	-	-	-	-

H=Household (includes only households with study participants shedding *S*. Typhi after the index case was treated)

* Study participants with gallstones

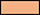
 Shaded cells indicate the month during which *S*. Typhi shedding was detected.

In bold is the number of days between the day the index case was diagnosed with typhoid fever and the day/s shedding was detected.

Households in bold (shaded 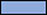) are those with individuals shedding multidrug resistant *S*. Typhi-Sample not collected

-Sample not collected

**Table 2.** Resistance patterns of *S*. Typhi isolated from index cases and household contacts

	Antimicrobials Resistance patterns	Index Cases		Household Contacts
		N (%)		N (%)
MDR	MDR + nalidixic acid resistance and reduced susceptibility to ciprofloxacin	11/32(34.3)		1/5(20)
	MDR + nalidixic acid and tetracycline resistance and reduced susceptibility to ciprofloxacin	1/32(3.1)		-
	MDR	3/32(9.3)		-
Non-MDR	Nalidixic acid resistance and reduced susceptibility to ciprofloxacin	3/32(9.3)		1/5(20)
	Reduced susceptibility to nalidixic acid and ciprofloxacin	5/32(15.6)		2/5(40)
	Reduced susceptibility to ciprofloxacin	2/32(6.2)		1/5(20)
	Susceptible to all drugs tested	7/32(21.8)		-

MDR= *S*. Typhi resistant to ampicillin, co-trimoxazole and chloramphenicol

Figures indicate the number/percentage of participants infected by *S*. Typhi exhibiting observed resistance patterns.

## Abbreviations

MDR: multidrug resistant
OD: optical density
SEM: standard error of mean
M: mean
SD: standard deviation
CI: confidence interval

## References

[1] Gonzalez-Escobedo G, Marshall JM, Gunn JS. (2011) Chronic and acute infection of the gall bladder by *Salmonella* Typhi: understanding the carrier state. Nature Reviews Microbiology.9(1), 9–14. 21113180 10.1038/nrmicro2490 PMC3255095

[2] Parkhill J, Dougan G, James K, (2001) Thomson N, Pickard D, Wain J, Complete genome sequence of a multiple drug resistant *Salmonella* enterica serovar Typhi CT18. Nature. 413 (6858), 848–52. 11677608 10.1038/35101607

[3] Kim CL, Cruz Espinoza LM, Vannice KS, (2022) The burden of typhoid fever in sub-Saharan Africa: a perspective. Research and Reports in Tropical Medicine. 1–9. 35308424 10.2147/RRTM.S282461 PMC8932916

[4] World Health Organization. Typhoid 2018. Available from: https://www.who.int/news-room/fact-sheets/detail/typhoid. Accessed March 2024.

[5] Dewan AM, Corner R, Hashizume M, (2013) Typhoid fever and its association with environmental factors in the Dhaka metropolitan area of Bangladesh: a spatial and time-series approach. PLoS neglected tropical diseases.7(1), e1998. 23359825 10.1371/journal.pntd.0001998 PMC3554574

[6] Khan MI, Ochiai R, Soofi S, (2012) Risk factors associated with typhoid fever in children aged 2–16 years in Karachi, Pakistan. Epidemiology & Infection.140(4), 665–72. 21676350 10.1017/S0950268811000938

[7] Crump JA. (2019) Progress in typhoid fever epidemiology. Clinical Infectious Diseases. 68 (Supplement_1), S4–9. 30767000 10.1093/cid/ciy846 PMC6376096

[8] Srinivasan M, Sindhu KN, Giri S, (2021) *Salmonella* Typhi shedding and household transmission by children with blood culture-confirmed typhoid fever in Vellore, South India. The Journal of infectious diseases. 224(Supplement_5), S593–600. 35238362 10.1093/infdis/jiab409 PMC8892528

[9] Marineli F, Tsoucalas G, Karamanou M, (2013) Mary Mallon (1869–1938) and the history of typhoid fever. Annals of gastroenterology: quarterly publication of the Hellenic Society of Gastroenterology. 26(2), 132. PMC3959940 24714738

[10] Gal-Mor O (2018) Persistent infection and long-term carriage of typhoidal and nontyphoidal salmonellae. Clinical microbiology reviews. 32(1), 10–1128. 10.1128/CMR.00088-18 PMC6302356 30487167

[11] Gunn JS, Marshall JM, Baker S, (2014) Ryan ET. *Salmonella* chronic carriage: epidemiology, diagnosis, and gallbladder persistence. Trends in microbiology. 22(11), 648–55. 25065707 10.1016/j.tim.2014.06.007 PMC4252485

[12] Prouty A, Schwesinger W, Gunn J. (2002) Biofilm formation and interaction with the surfaces of gallstones by Salmonella spp. Infection and immunity. 70(5), 2640–9. 11953406 10.1128/IAI.70.5.2640-2649.2002 PMC127943

[13] Crawford RW, Gibson DL, Kay WW, (2008) Identification of a bile-induced exopolysaccharide required for Salmonella biofilm formation on gallstone surfaces. Infection and immunity. 76(11), 5341–9. 18794278 10.1128/IAI.00786-08 PMC2573354

[14] Hall-Stoodley L, Costerton JW, Stoodley P. (2004) Bacterial biofilms: from the natural environment to infectious diseases. Nature reviews microbiology. 2(2), 95–108. 15040259 10.1038/nrmicro821

[15] González JF, Alberts H, Lee J, (2018) Biofilm formation protects *Salmonella* from the antibiotic ciprofloxacin in vitro and in vivo in the mouse model of chronic carriage. Scientific reports. 8(1), 222. 29317704 10.1038/s41598-017-18516-2 PMC5760579

[16] Kariuki S, Dyson ZA, Mbae C, (2021) Multiple introductions of multidrug-resistant typhoid associated with acute infection and asymptomatic carriage, Kenya. Elife. 10, e67852. 34515028 10.7554/eLife.67852 PMC8494480

[17] Mutai WC, Muigai AW, Waiyaki P, (2018) Multi-drug resistant *Salmonella enterica* serovar Typhi isolates with reduced susceptibility to ciprofloxacin in Kenya. BMC microbiology.18, 1–5. 30428828 10.1186/s12866-018-1332-3 PMC6236932

[18] Park SE, Pham DT, Boinett C, (2018) The phylogeography and incidence of multi-drug resistant typhoid fever in sub-Saharan Africa. Nature communications. 9(1), 5094. 10.1038/s41467-018-07370-z PMC6269545 30504848

[19] World Health Organization. (2019) Typhoid vaccines: WHO position paper, March 2018–Recommendations. Vaccine. 37(2), 214–6. 29661581 10.1016/j.vaccine.2018.04.022

[20] Mbae C, Mwangi M, Gitau N, (2020) Factors associated with occurrence of salmonellosis among children living in Mukuru slum, an urban informal settlement in Kenya. BMC Infectious Diseases. 20, 1–12. 10.1186/s12879-020-05134-z PMC7302364 32552753

[21] Crawford RW, Rosales-Reyes R, Ramirez-Aguilar M de la L, (2010) Gallstones play a significant role in *Salmonella* spp. gallbladder colonization and carriage. Proceedings of the National Academy of Sciences. 107(9), 4353–8. 10.1073/pnas.1000862107 PMC2840110 20176950

[22] Monack DM. (2012) *Salmonella* persistence and transmission strategies. Current opinion in microbiology. 15(1), 100–7. 22137596 10.1016/j.mib.2011.10.013

[23] Marchello CS, Hong CY, Crump JA. (2019) Global typhoid fever incidence: a systematic review and meta-analysis. Clinical Infectious Diseases. 68(Supplement_2), S105–16. 30845336 10.1093/cid/ciy1094 PMC6405273

[24] Meiring JE, Shakya M, Khanam F, (2021) Burden of enteric fever at three urban sites in Africa and Asia: a multicentre population-based study. The Lancet Global Health. 9(12), e1688–96. 34798028 10.1016/S2214-109X(21)00370-3 PMC8609278

[25] Antillón M, Warren JL, Crawford FW, , (2017) The burden of typhoid fever in low- and middle-income countries: A meta-regression approach. PLOS Neglected Tropical Diseases. 11(2), e0005376. 28241011 10.1371/journal.pntd.0005376 PMC5344533

[26] Mogasale V, Maskery B, Ochiai RL, (2014) Burden of typhoid fever in low-income and middle-income countries: a systematic, literature-based update with risk-factor adjustment. The Lancet Global Health. 2(10), e570–80. 25304633 10.1016/S2214-109X(14)70301-8

[27] Smith SM, Palumbo PE, Edelson PJ. (1984) *Salmonella* strains resistant to multiple antibiotics: therapeutic implications. The Pediatric Infectious Disease Journal. 3(5), 455. 10.1097/00006454-198409000-00017 6387645

[28] Shrestha KL, Pant ND, Bhandari R, (2016) Re-emergence of the susceptibility of the Salmonella spp. isolated from blood samples to conventional first line antibiotics. Antimicrob Resist Infect Control. 5(1), 22. 27231547 10.1186/s13756-016-0121-8 PMC4881163

[29] Ochieng C, Chen JC, Osita MP, (2022) Molecular characterization of circulating Salmonella Typhi strains in an urban informal settlement in Kenya. PLOS Neglected Tropical Diseases. 16(8), e0010704. 36007074 10.1371/journal.pntd.0010704 PMC9451065

[30] Butler T (2011) Treatment of typhoid fever in the 21st century: promises and shortcomings. Clinical Microbiology and Infection. 17(7), 959–63. 21722249 10.1111/j.1469-0691.2011.03552.x

[31] Senthilkumar B, Prabakaran G. (2005) Multidrug resistant *salmonella* Typhi in asymptomatic typhoid carriers among food handlers in Namakkal district, Tamil Nadu. Indian Journal of Medical Microbiology. 23(2), 92–4. 15928436 10.4103/0255-0857.16046

[32] Staley C, Weingarden AR, Khoruts A, (2017) Interaction of gut microbiota with bile acid metabolism and its influence on disease states. Applied microbiology and biotechnology. 101, 47–64. 27888332 10.1007/s00253-016-8006-6 PMC5203956

[33] Jahan F, Chinni SV, Samuggam S, (2022) The complex mechanism of the *Salmonella* Typhi biofilm formation that facilitates pathogenicity: a review. International Journal of Molecular Sciences. 23(12), 6462. 35742906 10.3390/ijms23126462 PMC9223757

[34] Lammert F, Gurusamy K, Ko CW, (2016) Gallstones. Nature Reviews Disease Primers. 2, 16024. 10.1038/nrdp.2016.24 27121416

[35] Enochsson L, Thulin A, Österberg J, (2013) The Swedish Registry of Gallstone Surgery and Endoscopic Retrograde Cholangiopancreatography (GallRiks): A Nationwide Registry for Quality Assurance of Gallstone Surgery. 148(5), 471–8. 10.1001/jamasurg.2013.1221 23325144

[36] Hatta M, Pastoor R, Scheelbeek PFD, (2011) Multi-Locus Variable-Number Tandem Repeat Profiling of *Salmonella enterica* Serovar Typhi Isolates from Blood Cultures and Gallbladder Specimens from Makassar, South-Sulawesi, Indonesia. PLOS ONE. 6(9), e24983. 21949819 10.1371/journal.pone.0024983 PMC3174255

[37] Dutta U, Garg PK, Kumar R, Tandon RK. Typhoid carriers among patients with gallstones are at increased risk for carcinoma of the gallbladder. Am J Gastroenterol. 2000 Mar;95(3):784–7. 10710075 10.1111/j.1572-0241.2000.01860.x

[38] Di Domenico EG, Cavallo I, Pontone M, Toma L, Ensoli F. Biofilm Producing Salmonella Typhi: Chronic Colonization and Development of Gallbladder Cancer. International Journal of Molecular Sciences. 2017 Sep;18(9):1887. 28858232 10.3390/ijms18091887 PMC5618536

[39] Devaraj A, González JF, Eichar B, (2021) Enhanced biofilm and extracellular matrix production by chronic carriage versus acute isolates of Salmonella Typhi. PLoS pathogens. 17(1), e1009209. 33465146 10.1371/journal.ppat.1009209 PMC7815147

[40] Eguale T, Marshall J, Molla B, (2014) Association of multicellular behaviour and drug resistance in *Salmonella enterica* serovars isolated from animals and humans in Ethiopia. Journal of Applied Microbiology. 117(4), 961–71. 24934091 10.1111/jam.12579 PMC4165716

[41] Tabak M, Scher K, Chikindas ML, (2009) The synergistic activity of triclosan and ciprofloxacin on biofilms of *Salmonella* Typhimurium. FEMS Microbiology Letters. 301(1), 69–76. 19843306 10.1111/j.1574-6968.2009.01804.x

[42] Thaver D, Zaidi AKM, Critchley J, (2009) A comparison of fluoroquinolones versus other antibiotics for treating enteric fever: meta-analysis. BMJ. 338, b1865. 19493939 10.1136/bmj.b1865 PMC2690620

