# Supplementary material for: Fecal Shedding, Antimicrobial Resistance and In Vitro Biofilm formation on Simulated Gallstones by Salmonella Typhi Isolated from Typhoid Cases and Asymptomatic Carriers in Nairobi, Kenya
Source: Int J Clin Microbiol. Author manuscript; Available in PMC 2024 Sep 24. (PMC11421374; doi:10.14302/issn.2690-4721.ijcm-24-5030)
Supplement: Supplementary Table S1 [file NIHMS2019222-supplement-Supplementary_Table_S1.docx]

| **Household** | **Antibiotic given** | **Duration of Course of Antibiotics**  **(Days)** |
| --- | --- | --- |
| H1 | Co-amoxiclav | 5 |
| H2 | Co-amoxiclav | 5 |
| H3 | Co-amoxiclav | 7 |
| H4 | Pylokit (lansoprazole, tinidazole and clarithromycin) | 14 |
| H5 | Ciprofloxacin | 5 |
| H6 | Co-amoxiclav | 14 |
| H7 | Ciprofloxacin | 5 |
| H8 | Co-amoxiclav | 14 |
| H9 | Cefuroxime | 5 |
| H10 | Co-amoxiclav | 5 |
| H11 | Co-amoxiclav | 14 |
| H12 | Ciprofloxacin | 5 |
| H13 | Ceftriaxone (Injection) | 1 |
|  | Azithromycin | 5 |
|  | Gentamycin | 5 |
| H14 | Ciprofloxacin | 5 |
|  | Azithromycin | 7 |
| H15 | Co-amoxiclav | 14 |
| H16 | Ciprofloxacin | 10 |
| H17 | Co-amoxiclav | 14 |
| H18 | Ceftriaxone (Injection) | 1 |
|  | Levofloxacin | 5 |
| H19 | Ciprofloxacin | 5 |
| H20 | Co-amoxiclav | 14 |
| H21 | Co-trimoxazole | 5 |
|  | Azithromycin | 6 |
|  | Cefuroxime | 7 |
| H22 | Cefuroxime | 5 |
| H23 | Co-amoxiclav | 14 |
| H24 | Co-trimoxazole | 5 |
| H25 | Azithromycin | 7 |
|  | Co-trimoxazole | 5 |
| H26 | Ciprofloxacin | 7 |
| H27 | Ciprofloxacin | 5 |
| H28 | Ciprofloxacin | 5 |
| H29 | Ciprofloxacin | 5 |
|  | Amoxicillin-clavulanate | 7 |
| H30 | Ciprofloxacin | 5 |
| H31 | Co-trimoxazole | 5 |
| H32 | Ciprofloxacin | 5 |
|  |  |  |

**Supplementary Table S1**. Antibiotics given to typhoid fever index cases.
