# Supplementary material for: Fecal Shedding, Antimicrobial Resistance and In Vitro Biofilm formation on Simulated Gallstones by Salmonella Typhi Isolated from Typhoid Cases and Asymptomatic Carriers in Nairobi, Kenya
Source: Int J Clin Microbiol. Author manuscript; Available in PMC 2024 Sep 24. (PMC11421374; doi:10.14302/issn.2690-4721.ijcm-24-5030)
Supplement: Supplementary Table S2 [file NIHMS2019222-supplement-Supplementary_Table_S2.docx]

|  | **Index Cases** | |  | **Household Contacts**  **(Age and Gender)** | | |
| --- | --- | --- | --- | --- | --- | --- |
|  | **Age and Sex** | ***S*. Typhi Positive Sample** | **Contact 1** | | **Contact 2** | **Contact 3** |
| H1 | 45 years 1 month, Female | Stool | - | | - | - |
| H2 | 26 years 2 months, Female | Blood | 32 years, Male | | - | - |
| H3 | *45 years, Female | Blood and Stool | - | | - | - |
| H4 | 38 years 5 months, Female | Blood | 12 years, 11 months, Male | | 44 years 6 months, Male | - |
| H5 | 34 years 8 months, Female | Stool | 28 years, Female | | - | - |
| H6 | *22 years, Female | Stool | *38 years, Male | | - | - |
| H7 | *22 years 7 months, Male | Blood and Stool | 22 years 11 months, Male | | *20 years 1 months, Male | - |
| H8 | *32 years 11 months, Female | Stool | 35 years, Male | | - | - |
| H9 | *33 years 6 months, Male | Blood and Stool | 26 years, Female | | - | - |
| H10 | 56 years 11 months, Female | Stool | - | | - | - |
| H11 | 32 years, Male | Stool | 27 years, Female | | - | - |
| H12 | 29 years 10 months, Male | Blood and Stool | - | | - | - |
| H13 | 23 years 7 months, Female | Blood and Stool | - | | - | - |
| H14 | 18 years 2 months, Male | Blood | *20 years 4 months, Male | | 37 years 6 months, Female | - |
| H15 | 12 years 4 months, Male | Stool | 32 years, Female | | - | - |
| H16 | 24 years 2 months, Male | Blood and Stool | 13 years, Female | | - | - |
| H17 | *34 years 3 months, Male | Blood and Stool | 28 years 3 months, Female | | - | - |
| H18 | 45 years 5 months, Female | Blood and Stool | - | | - | - |
| H19 | 29 years 11 months, Male | Blood and Stool | - | | - | - |
| H20 | 26 years 11 months, Female | Blood | - | | - | - |
| H21 | 13 years 4 months, Female | Blood and Stool | 38 years, Female | | *39 years 11 months, Male | - |
| H22 | 50 years 5 months, Male | Stool | - | | - | - |
| H23 | *31 years, Male | Blood | 29 years, Female | | - | - |
| H24 | 13 years, Male | Blood | 46 years, Male | | 43 years, Female | 23 years, Female |
| H25 | 25 years 7 months, Female | Stool | 30 years, Male | | - | - |
| H26 | 12 years 3 months, Female | Blood and Stool | 39 years, Female | | - | - |
| H27 | 22 years 9 months, Male | Blood | 33 years 6 months, Male | | 13 years, Male | 25 years, Female |
| H28 | *45 years 3 months, Female | Stool | 49 years 9 months, Male | | 23 years 8 months, Male | - |
| H29 | *29 years, Male | Blood and Stool | *30 years, Female | | - | - |
| H30 | 29 years 2 months, Male | Blood and Stool | 19 years 9 months, Male | | - | - |
| H31 | 22 years 3 male, Female | Stool | 21 years, Male | | 27 years, Male | - |
| H32 | 30 years 5 months, Male | Stool | 25 years, Male | | - | - |
| Listed samples are culture positive samples from index cases collected before medication.  H=Household  *Cases shedding *S*. Typhi after treatment and household contacts shedding the bacteria during follow-up period.  Data represents only cases followed up after treatment and their household contacts (all >12 years old) | | | | | | |

**Supplementary Table S2**. Index Cases and Household Contacts
